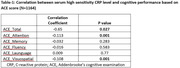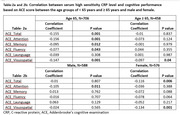# The correlation between inflammatory marker and cognitive performance in TATA Longitudinal Study of Aging cohort

**DOI:** 10.1002/alz.092566

**Published:** 2025-01-09

**Authors:** Prathima Arvind, Abhishek M L, Albert Stezin, Latha Diwakar, Amitha C M, Ajith Partha, Rajitha Narayanasamy, Divya N M, Meghana R, Meenakshi Menon, Sunitha HS, Goutham Velavarajan, Palash K Malo, Sadhana Singh, Vindhya Vishwanath, Dev Kumar HS, Shafeeq K Shahul Hameed, Jonas S. Sundarakumar, Thomas Gregor Issac

**Affiliations:** ^1^ Centre for Brain Research, Indian Institute of Science, Bangalore, Karnataka India; ^2^ Centre for Brain Research, Indian Institute of Science, Bengaluru, Karnataka India

## Abstract

**Background:**

In the scientific literatures, C‐reactive protein (CRP), a well‐known inflammatory biomarker^1^, has been closely examined in connection to cognitive function^2^. However, less knowledge on correlation study between the serum CRP level and different cognitive domain. Therefore, we aimed to study the relation between CRP level and cognition performance in TLSA cohort.

**Method:**

The study involved 1164 participants from the TLSA cohort, an ongoing longitudinal study on aging in Indian urban populations. Cognitive performance was tested using ACE‐III and evaluated CRP levels. Spearman's correlation analysis was performed to see the relation between the CRP level and cognitive performance in whole cohort as well as to identify gender and age‐specific associations between inflammation and cognitive function.

**Result:**

The mean age of the entire cohort was 61.72 ± 9.39 (N=1164), with a gender distribution of 50.5% male and 49.5% female. The median education level stood at 14.5, with a standard deviation (SD) of ± 4.26. Notably, ACE‐total, attention, and visuospatial domains exhibited significant inverse associations with CRP levels across the entire cohort (Table 1). In the subgroup analysis, individuals below 65 years of age showed significant inverse associations between CRP levels and ACE‐total, attention, memory, fluency, and visuospatial domains. However, in the above 65 years age group, only ACE‐Visuospatial demonstrated an inverse association with CRP levels (Table 2a). Further stratification by gender revealed ACE‐attention to be inversely associated in male participants, while ACE‐total and visuospatial domains exhibited associations in the female group (Table 2b).

**Conclusion:**

This study reveals significant inverse connections between CRP levels and cognitive domains, particularly in the younger population, suggesting potential hormonal influences. The findings underscore the need for tailored approaches to understand the complex interplay between inflammation and cognitive function across diverse demographic contexts.